# Comparison of single and two-tunnel techniques during open treatment of acromioclavicular joint disruption

**DOI:** 10.1186/1471-2482-14-53

**Published:** 2014-08-15

**Authors:** Zhiyong Hou, Jove Graham, Yingze Zhang, Kent Strohecker, Daniel Feldmann, Thomas R Bowen, Wei Chen, Wade Smith

**Affiliations:** 1 Department of Orthopaedic Surgery, Third Hospital of Hebei Medical University, Shijiazhuang, Hebei 050051, China; 2 Department of Orthopaedic Surgery, Geisinger Medical Center, Danville, PA 17822, USA; 3 Mountain Orthopaedic Trauma Surgeons at Swedish, 701 East Hampden Avenue Suite 515, Englewood, CO 80113, USA

**Keywords:** Acromioclavicular joint, Single-tunnel, Two-tunnel, Reconstruction, Augmentation

## Abstract

**Background:**

Coracoclavicular (CC) ligament reconstruction with semitendinosus tendon (ST) grafts has become more popular and has achieved relatively good results; however optimal reconstruction technique, single-tunnel or two-tunnel, still remains controversial. This paper is to compare the clinical and radiographic data of allogenous ST grafting with single- or two-tunnel reconstruction techniques of the AC joint.

**Methods:**

The outcomes of 21 consecutive patients who underwent anatomical reduction and ST grafting for AC joint separation were reviewed retrospectively. Patients were divided into two groups: single-tunnel group (11) and two-tunnel group (10). All patients were evaluated clinically and radiographically using a modified UCLA rating scale.

**Results:**

The majority of separations (18 of 21) were Rockwood type V, with one each in type III, IV and VI categories. The overall mean follow-up time was 16 months, and at the time of the latest follow-up, the overall mean UCLA rating score was 14.1 (range 8–20).

The percentage of good-to-excellent outcomes was significantly higher for patients with the two-tunnel technique than for those with the one-tunnel technique (70% vs. 18%, respectively, p = 0.03). Within the single-tunnel group, there was no statistically significant difference in percentage of good-to-excellent outcomes between patients with vs. without tightrope augmentation (17% vs 20%, p > 0.99). Similarly, within the two-tunnel group, there was no significant difference in the percentage of good-to-excellent outcomes between the graft only and augment groups (67% vs. 75%, p > 0.99).

**Conclusion:**

Anatomical reduction of the AC joint and reconstruction CC ligaments are crucial for optimal joint stability and function. Two-tunnel CC reconstruction with an allogenous ST graft provides superior significantly better radiographic and clinical results compared to the single-tunnel reconstruction technique.

## Background

Acromioclavicular (AC) joint injuries are among the most commonly occurring problems in the young and active patient population. Higher-grade AC joint injuries (Rockwood types III through VI) represent failure of the coracoclavicular (CC) ligament complex, which is formed by the conoid and trapezoid ligaments. This complex has been termed the primary suspensory structure of the upper limb [1,2]. In the literature, the incidence of traumatic AC joint separation varies from 3 to 4 per 100,000 people with 25-52% of these occurring during sporting activities, and they are also one of the most common shoulder injuries seen in orthopaedic traumatology [2-5]. For certain Rockwood type III AC joint separations and all type IV, V, and VI injuries, surgical treatment has been recommended to prevent disabling pain, weakness, and deformity [6-8]. Although more than 60 surgical techniques have been reported, the frequency of failure to maintain reduction after surgical treatment remains high [9,10].

Recently, CC ligament reconstruction with tendon grafts has become more popular and has achieved relatively good results [11,12]. Biomechanical studies focusing on an anatomic reconstruction of the CC ligament complex using tendon grafts have reported promising potential for this technique [13-15]. Semitendinosus tendon (ST) grafting and anatomic reconstruction can be imitated, providing stability to the clavicle that is very close to that provided by the intact ligaments [13]. However, optimal reconstruction technique, single-tunnel or two-tunnel, still remains controversial. Anatomical two-tunnel reconstruction with tendon grafts or synthetic materials seems appealing because it has been shown by biomechanical studies to restore the original two ligaments (the conoid and trapezoid) and to produce an ultimate failure load that is equivalent to that of native CC ligaments [13-15]. However, it is technically difficult and theoretically increases the risk of fracture [16].

The purpose of this retrospective study was to analyze the clinical and radiographic data of allogenous ST tendon grafting with single- or two-tunnel reconstruction techniques of the CC ligaments. We hypothesize that anatomic reconstruction of the AC joint disruption using two-tunnel reconstruction technique results in a satisfying clinical function and provides stable fixation.

## Methods

**Figure 1:**
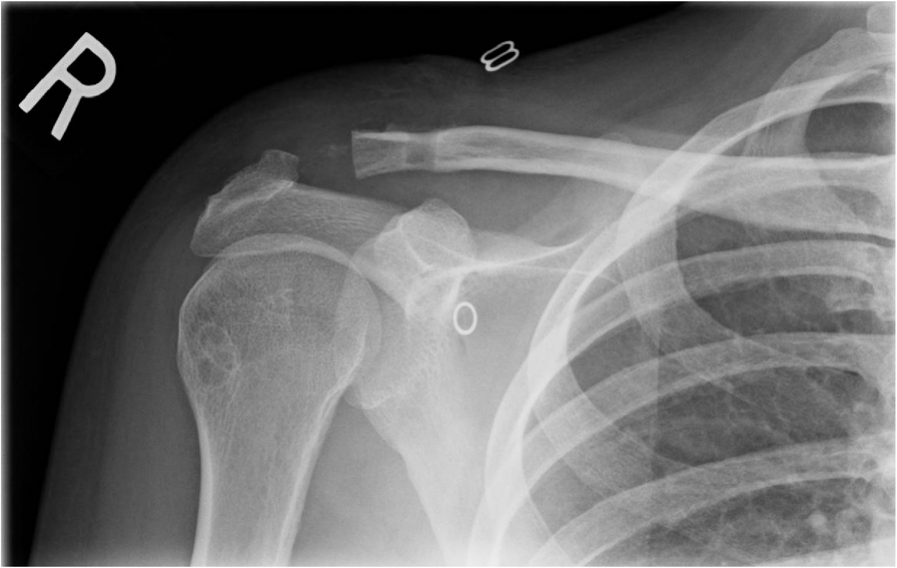
ST allograft reconstruction of the AC joint with single-tunnel technique.

**Figure 2:**
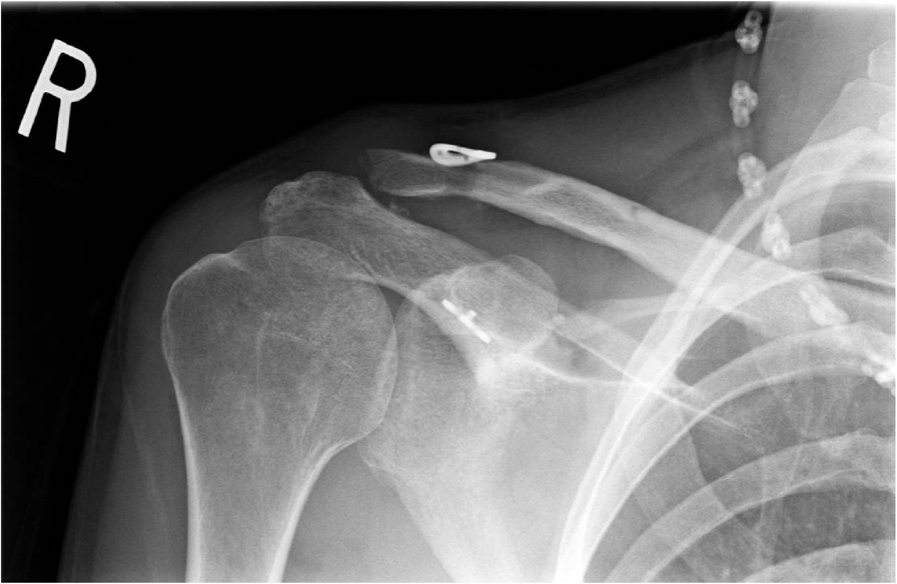
ST Allograft with tightrope augment reconstruction of the CC joint with single-tunnel technique.

**Figure 3:**
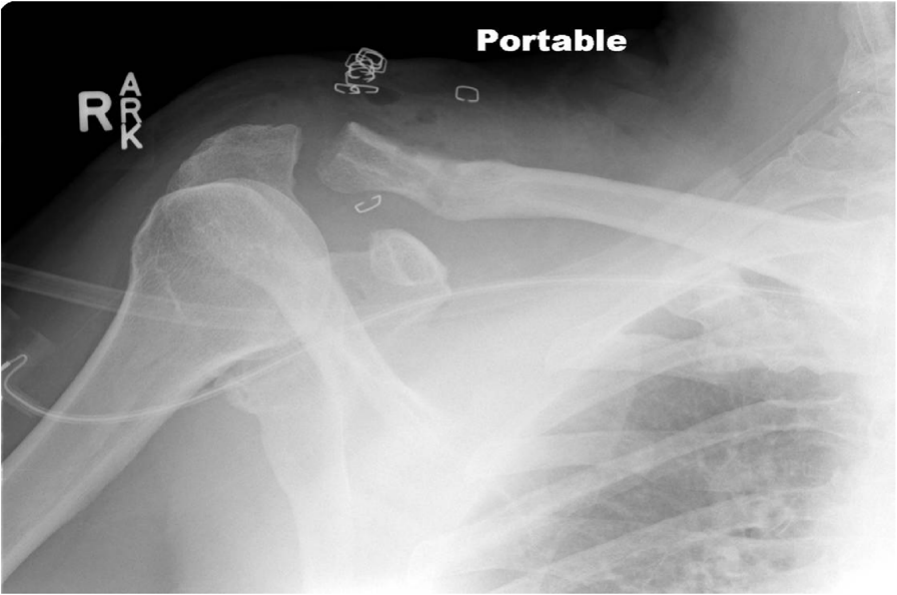
ST allograft reconstruction of the CC joint with two-tunnel technique.

**Figure 4:**
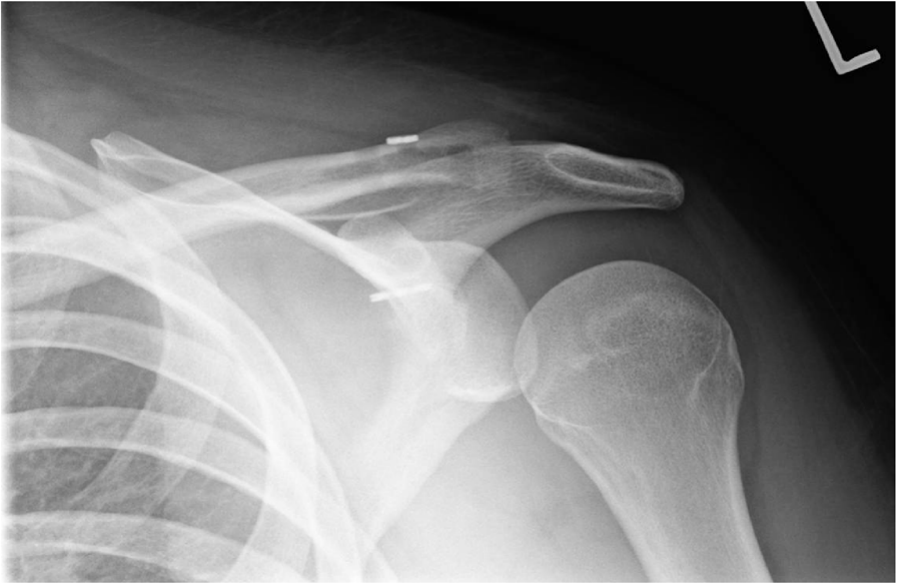
ST Allograft with tightrope augment reconstruction of the CC joint with two-tunnel technique.

Between June 2003 and January 2009, twenty-three patients underwent open operation for AC joint reconstruction with ST allograft at our institution. In the earlier study period before 2007, we mostly used single-tunnel technique, and after 2007 mostly the two-tunnel technique. For analysis we divided patients into two groups: single-tunnel group and two-tunnel group. Patient data were collected retrospectively, including gender, age at the time of surgery, injury mechanism, classification according to Rockwood, and surgical technique. Patients with at least 12 months of clinical follow-up were included in this study. Patients were excluded if they had a previous shoulder injury, arthritis, or an associated neurological deficit on the side of injury.The procedure was performed with the patient in the beach chair position under general anesthesia in combination with an interscalene block. An anterior deltopectoral approach was utilized with saber incision, The AC joint, the lateral end of the clavicle, and the coracoid process were exposed. Subperiosteal detachment of the deltotrapezial fascia from the clavicle was performed. The distal end of the clavicle was resected 8 to 10 mm using an oscillating saw. For the single-tunnel technique, a 6-mm drill hole was made about 1.5-2 cm medial to the remaining end of the clavicle superior to inferior in a 300 posterior to anterior angle. A ST allograft was prepared by placing a whipstitch (Arthrex #2 Fiberwire suture, Naples, FL, USA) on either end. After reducing the distal clavicle down to the acromion anatomically, the ST graft was introduced around the base of the coracoid and then both ends of the graft up through the clavicle hole. The graft was then mechanically tensioned and a 5.5 mm Bio-tenodesis screw was placed down through the center of the ST graft fixing it to the clavicle. The free ends of the graft were then passed underneath the clavicle and tied to themselves for additional fixation (Figure 1). If using a tightrope augment (Arthrex Fiberwire No. 5, Naples, FL, USA), a guide was used to place a pin from a point medial to the lateral tunnel, to the base of the coracoid. A 4.5 mm reamer was then used to create a tunnel through the clavicle and coracoid. The tight rope device was placed through the clavicular and then coracoid tunnel and endobutton secured against inferior cortex of coracoid. The tight-rope was then tied after fixation of the graft. Later in the series, a single clavicular tunnel was utilized for both the graft and tight-rope. The graft was placed around the coracoid and through the clavicular tunnel and tightrope device (Figure 2).For the two-tunnel technique, the same delto-pectoral approach was used. Two holes were drilled in the clavicle to reconstruct each of the two CC ligaments, trapezoid and conoid ligaments. The lateral tunnel is created as in the single-tunnel technique. The medial tunnel is located 4.5 cm medial to the AC joint. A 5.5 mm tunnel is reamed like the medial tunnel. A single ST graft was prepared and looped under the coracoid. The lateral free end was brought up through the lateral tunnel, and the medial free end through the medial tunnel. The AC joint is reduced, and the grafts fixed into the tunnels with 5 mm biotenodesis screws and the graft tied to itself (Figure 3). If using tightrope augment, a guide pin is placed between the two graft tunnels, from midline, through the clavicle and base of coracoid. A 4.5 mm tunnel is reamed over the guide wire and the Tight-rope device placed through the clavicle and coracoid and secured to the inferior cortex of the coracoid. The device is tightened and tied after graft fixation (Figure 4). After reconstruction, attention was directed to repair of the deltotrapezial interval. This was performed in a pants-over-vest fashion using #1 or #2 non-absorbable sutures in an interrupted fashion. A layered closure was then performed. A drain was not utilized.

All patients were placed in a sling immobilizer post-op for 4 to 6 weeks. Gentle pendulums and Codman’s were begun post-op day 1. At 4 weeks therapy was begun with passive motion and cuff isometrics. Resistive program started at 8 weeks. Patients were generally allowed to return to manual work and athletics at 4 to 6 months depending on level of rehabilitation. Contact sports not prior to six months. All patients were evaluated clinically and radiographically using a modified UCLA rating scale [5,17], which reflects three parts: maintenance of reduction, objective evaluation of the patient’s function, and complications secondary to operation. In the radiological evaluation, the roentgenographic rating was determined by the degree of displacement of the AC joint, which was evaluated by measuring the relation between the acromion and the clavicle on the anteroposterior view for vertical displacement (reduced = 4 points, subluxed = 2 points, dislocated = 0 points). In the physical evaluation, range of motion (ROM), pain, weakness, and complications were recorded. Finally, patients were asked their overall satisfaction with the postoperative result, with 0 points for dissatisfaction or unsure and 2 points for satisfaction.

Table 1 shows the relative weight given to each category of the rating scale and describes the criteria by which a patient was assigned an overall final result of excellent, good, fair, or poor.

**Table 1:** **The modification of the UCLA rating scale**^**8.17**^

**Category**	**Points**
Maintenance of reduction	
Reduced	4
Subluxion	2
Dislocation	0
Range of motion	
Full	2
Improved from preoperative	1
No change from preoperative	0
Strength	
Normal	2
Improved from preoperative	1
Unimproved from preoperative	0
Pain	
None	4
With strenuous activity	3
With moderate activity	2
With mild activity	1
All the time	0
Weakness	
None	2
With strenuous activity	1
All the time	0
Change in occupation	
Same or more strenuous	2
Less strenuous	0
Complication	
None	2
Minor/resolved	1
Major/affected outcome	0
Patient satisfaction	
Yes	2
No or unsure	0

Results: excellent, 18–20; good, 15–17; fair, 12–14; poor, ≤ 11.

Percentages of good-to-excellent outcomes and maintenance of reduction (reduced or subluxed) were compared between the two reconstruction procedures (single vs. two-tunnel), and between augmentation techniques (with vs. without tightrope). Because of the relatively small sample sizes, Fisher’s exact test was used in place of chi-square testing at a significance level of p < 0.05. All analysis was performed using SAS statistical software (SAS 9.2, Cary, NC). Waiver of patient consent was granted by Institutional Review Board of Geisinger Medical Center for retrospective chart review.

## Results

From the initial 23 patients who were surgically treated, two patients were lost to follow up and were excluded. Table 2 summarizes the demographics and injury characteristics of the 21 patients remaining in the study. The majority of fractures (18 of 21) were Rockwood type V, with one fracture each in type III, IV and VI categories. Most of those patients had received primary unsuccessful conservative care and switched to operative management, and one patient underwent a failed Weaver-Dunn procedure.

**Table 2:** Demographic and injury characteristics, by single-tunnel and two-tunnel group

**Parameter**	**Single-tunnel (11)**	**Two-tunnel (10)**
Gender		
Male	6	9
Female	5	1
Mean age (range), years	37 (20–55)	42 (20–63)
Side of Fracture		
Right	5	8
Left	6	2
Mechanism of Injury		
Sporting	6	6
Traffic accident	4	2
Fall	1	2
Rockwood Classification		
C3	1	0
C4	0	1
C5	10	8
C6	0	1
Mean length of follow up (range), months	16 (12–38)	15 (12–40)

The overall mean follow-up time was 16 months, and at the time of the latest follow-up, the overall mean UCLA rating score was 14.1 (range 8–20). Eleven (52%) patients rated the outcome as good to excellent, 3 (14%) rated it as fair, and 7 (33%) rated it as poor. Three of 21 patients underwent additional revision surgery for the failed CC ligament repair or reconstruction.

Of the 21 patients, eleven patients underwent allogenous ST grafting with single-tunnel reconstruction technique, and 6 of these received tightrope augmentation. Ten patients underwent allogenous ST grafting with two-tunnel reconstruction technique: four of these received one ST graft plus one tightrope graft (“ST-tightrope”), while the other six received two ST grafts (“ST-ST”).

Table 3 summarizes the UCLA rating scale scores at last follow-up for the two groups (single- and two-tunnel), subdivided by augmentation type. The percentage of good-to-excellent outcomes was significantly higher for patients with the two-tunnel technique than for those with the one-tunnel technique (70% vs. 18%, respectively, p = 0.03). Within the single-tunnel group, there was no statistically significant difference in percentage of good-to-excellent outcomes between patients with vs. without tightrope augmentation (17% vs 20%, p > 0.99). Similarly, within the two-tunnel group, there was no significant difference in the percentage of good-to-excellent outcomes between ST-tightrope and ST-ST patients (75% vs. 67%, p > 0.99).

**Table 3:** Number of patients receiving single-tunnel vs. two-tunnel techniques, subdivided by augmentation type, with clinical outcome results based on modification of the UCLA rating scale

**UCLA rating scale**	**Single-tunnel (n = 11)**		Two-tunnel (n = 10)^*^	
	**With augment**	**Without augment**^**^**^	**ST-tightrope**	**ST-ST**^**#**^
Excellent	1	1	1	2
Good	0	0	2	2
Fair	2	1	1	1
Poor	3	3	0	1
Total	6	5	4	6
N(%) with excellent or good	1 (17%)	1 (20%)	3 (75%)	4 (67%)

*Two-tunnel group had significantly higher percentage of good-to-excellent outcomes than single-tunnel group, p = 0.03.

^^^No significant difference between with vs. without augmentation for single-tunnel group, p > 0.99.

^#^No significant difference between ST-tightrope vs. ST-ST for two-tunnel group, p > 0.99.

We noted that complications were observed in three of the 21 patients: two patients in the two-tunnel group had infection, and one patient in the single-tunnel group had a coracoid fracture. Calcification of the CC ligament occurred in one case, but it did not appear to cause symptoms, and was therefore not considered a complication. No patient had neurovascular or post-traumatic arthritis of the injured AC joint.

## Discussion

Our data demonstrated that allogenous ST grafting with two-tunnel reconstruction technique of the AC joint yielded excellent or good clinical outcomes more frequently compared to single-tunnel reconstruction technique. These results also suggest that the materials used for augmentation in the two-tunnel reconstruction technique do not impact the clinical result. In this technique, one ST allograft combined with one tightrope graft construction can provide similar outcomes to using ST allograft in both tunnels. We also saw no significant differences between patients with and without tightrope augment in the single-tunnel technique group.

Based on well established anatomical ligament reconstruction in the knee injury, reconstructing the CC ligament using tendon graft for AC joint injury has become more popular because the construct is more physiologic, does not require implant removal and preserves the CA ligament [18,19]. ST tendon grafts are most common used for this procedure, which can be either autografts or allografts, and have achieved relatively good results [11-13,20,21]. The harvesting of an autogenous tendon may not result in long-term functional impairment but may still cause some morbidity associated with the donor site, and also create a second operative site during AC joint surgery [22]. Nicholas et al. [12] achieved excellent outcomes after fresh-frozen ST allograft reconstruction of the CC ligament; patients reported significant pain relief, return of normal strength and function, negligible loss of motion, and no loss of reduction on postoperative radiographs. Based on this information, the substitution of allograft material has become a routine procedure in our institution. The current surgical technique for the CC ligament reconstruction can be graft tendon passed though the clavicle with single tunnel or two tunnels technique [16,23], looped around the base of the coracoids [24], passed through a transosseous tunnel in the coracoids [25], or fixed to the base of coracoid using an anchor technique [6]. The CC ligament is stabilized by 2 sets of ligamentous structures: the conoid and trapezoid. Single-tunnel or two-tunnel reconstruction still remains controversial. Mazzocca et al. considered that each CC ligament has a separate function, and so each must be considered in reconstructive procedures [26]. Anatomical two-tunnel reconstruction with tendon grafts has yielded good results because it restores the original 2 ligaments and produces an ultimate strength that is equivalent to that of native CC ligaments [14,15,23]. However, two-tunnel techniques are technically difficult, with increased risk of fracture, and sometimes are not possible in patients with a small clavicle [13,16]. This technique should be performed by an experienced arthroscopist [23]. Yoo et al. [16] reported that single-tunnel reconstruction has some advantages over two-tunnel techniques. They reconstructed CC ligaments in 21 patients using a single-tunnel ST autograft and achieved superior clinical result. 17 (81%) of the 21 patients maintained complete reduction, and only 1 patient (reportedly a manual laborer) had complete reduction loss. In our cohort, there was a statistically significant difference in percentages of good-to-excellent UCLA scores between the single-tunnel and two-tunnel groups. The two-tunnel group had better scores, with the caveat that we observed two cases of infection in the two-tunnel group which may be related to the greater length and complexity of this procedure as compared to the single-tunnel technique.

Anatomical two-tunnel reconstruction with ST tendon grafts or synthetic materials provided similar results. The tightrope system, consisting of one round clavicle titanium button and one long coracoid titanium button connected by non-absorbable sutures (No. 5 Ethibond suture), has been initially utilized for repair of acute syndesmosis disruptions. The application has been extended and previously described for AC joint dislocations [27,28]. It can be used as a single graft device or an augment for the other tendon graft construction. Two-tunnel reconstruction technique has been shown by biomechanical studies to restore the strength of the original two ligaments (the conoid and trapezoid) and result in significantly higher stability in the superoinferior as well as the anteroposterior plane when compared with the native CC ligaments [11,14,15,29]. Grafting materials for the two-tunnel technique use are variable, and may include two tendon grafts, two tightrope grafts, or one tendon with one tightrope grafts. Salzmann et al. [23] reported on 23 consecutive patients with the acute AC joint disruption who underwent two-tunnel anatomical reconstruction of CC ligaments using two flip-button tightropes. This procedure yielded satisfactory clinical function and provided a stable fixation at intermediate-term follow-up. In our two-tunnel group, most patients had good-to-excellent UCLA scores at last followup, and this result did not vary between the cases treated with one ST graft and one tightrope graft versus those treated with two ST grafts.

Augmentation has been shown to be beneficial during CC ligament reconstructions by biomechanical studies [30,31]. An effective augmentation must have biomechanical properties enabling it to shield the repair or reconstruction from excessive tensile force, ideally allowing early rehabilitation. It seems desirable for an augmentation to possess strength and stiffness similar to those of the intact CC ligament complex, thus protecting against physiologic loads while allowing for physiologic motion between the clavicle and coracoid. Tienen et al. [32] had good results with using an open modified Weaver-Dunn technique and AC joint augmentation with absorbable, braided suture in 21 paptients. The tightrope augmentation was initially described for acute AC joint dislocation and represented an excellent biological augmentation technique by Hernegger [27]. Scheibel et al. [33] also reported using a gracilis tendon reconstruction augmented with a tightrope achieved good and excellent results and maintained good reduction for acute AC joint dislocations with one year follow up. Recently, Yoo et al. [16] also reported a superior result by using the tightrope augment technique to protect the ST graft though the same tunnel during the healing period. They considered the tightrope augment was really important factor for their successful surgical procedure and good outcomes. However, in our one-tunnel group, although the sample size was small, we saw no significant difference between patients treated with and without tightrope augmentation. Both of them had a higher re-dislocation rate and achieved the inferior results comparing to the two-tunnel group. From our results, we cannot definitively state that tightrope augmentation is not important and effective for the CC complex reconstruction, but our results do provide strong evidence that the reconstruction technique (specifically the choice between one or two tunnels) largely impacts the radiographic and clinical outcomes.

The principal limitations of this study are the relative small sample size who met our inclusion criteria and the fact that we did not have preoperative functional scores. Thus, our conclusions are focused on the substantial difference in success rates we saw between the single-tunnel and two-tunnel groups (18% vs. 70%), and we have limited ability to assess and compare other aspects of the procedures. In addition, because this was an observational study, our data did not permit an accurate assessment of the time to functional recovery. The two-tunnel technique became a standard technique at our institution at a later date than the single-tunnel technique, and so it is possible that surgeon experience may have played a role in the different outcomes among groups. However, we do not believe this confounding factor would be substantial enough to explain the large difference in the two groups that we observed.

## Conclusion

Anatomical reduction the AC joint and biomechanical reconstruction CC ligaments are crucial for the optimal joint stability and function. Two-tunnel CC reconstruction with an allogenous ST graft provides superior radiographic and clinical results compared to single-tunnel reconstruction technique.

## Abbreviations

CC ligament: Coracoclavicular ligament; ST tendon: Semitendinosus tendon; AC joint: Acromioclavicular joint; UCLA shoulder rating scale: University of California at Los Angeles shoulder rating scale; ROM: Range of motion.

## Competing interests

The authors declare that they have no competing interests.

## Authors’ contributions

ZH and WS designed research; JG, KS and WC analyzed data and performed statistical analysis. All authors read and approved the final manuscript.

## Pre-publication history

The pre-publication history for this paper can be accessed here:

http://www.biomedcentral.com/1471-2482/14/53/prepub
